# Medical futility and the ethics of continuing treatment: a hermeneutic inquiry into patient and physician perspectives

**DOI:** 10.1186/s13010-025-00200-3

**Published:** 2025-10-21

**Authors:** Ling-Lang Huang

**Affiliations:** School of Medicine, Mackay Medical University, New Taipei City, Taiwan

**Keywords:** Medical futility, Shared decision-making, Hermeneutics, Interpretative phenomenological analysis, Fusion of horizons

## Abstract

**Background:**

In the context of medically futile treatment, clinical decision-making often becomes ethically and existentially fraught, especially when physicians and patients navigate the space between prolonging life and preserving its meaning. Existing shared decision-making (SDM) models often rely heavily on empirical rationality, yet overlook the ontological depth of patient experience.

**Methods:**

Drawing on Heidegger’s concept of *being-in-the-world* and Gadamer’s *fusion of horizons*, we conducted an interpretative phenomenological analysis (IPA) of in-depth interviews with a terminal cervical cancer patient and three attending physicians (specialists in cardiology, cardiac surgery, and gynecologic oncology). These philosophical frameworks guided both the analytic lens and the ethical interpretation of SDM practices in contexts of medical futility.

**Results:**

Our findings reveal that decisions to continue aggressive treatment, even when medically futile, are not mere irrationalities. Rather, they emerge from divergent value orientations and temporal understandings between patients and physicians. A clinically “correct” decision may be ethically inadequate if it fails to integrate the patient’s lived horizon.

**Conclusions:**

We propose a hermeneutic framework for SDM that supplements the evidence-based model with three core steps: attunement to the patient’s existential situation, fusion of horizons between patient and physician, and respect for irreducible differences. This approach allows for ethically grounded decisions that honor both medical expertise and the patient’s being-in-the-world.

**Trial registration:**

Not applicable.

**Supplementary Information:**

The online version contains supplementary material available at 10.1186/s13010-025-00200-3.

## Background

When physicians determine that further curative treatment is medically futile, but patients continue to hope for life-prolonging interventions, how should clinical decisions ethically balance medical expertise with patients’ values?

In early 2021, Ms. M, a woman in her late forties, was diagnosed with stage I cervical cancer at Hospital (A) After receiving three rounds of chemotherapy, her tumor—approximately eight centimeters in diameter—did not shrink. By October, metastasis was found in her heart and left supraclavicular area. Hospital A concluded that further aggressive treatment was futile and recommended a transition to palliative care. However, unwilling to give up hope, Ms. M sought treatment at Hospital (B) Recognizing her desire to prolong life, physicians at Hospital B performed surgery to remove the tumor from her right ventricle, followed by additional chemotherapy. (All personal and institutional identifiers have been removed.)

This case not only illustrates the technical difficulties in determining medical futility but also highlights how divergent lifeworlds between physicians and patients complicate the practice of SDM.

To date, there remains no consensus regarding who should hold final authority over determinations of medical futility. Some scholars argue that such judgments should rest with physicians based on professional expertise and empirical evidence [1]. However, this position potentially conflicts with the ethical principle of respecting patient autonomy. Conversely, emphasizing the primacy of patients’ values and choices [2] may challenge the physician’s role as a moral agent responsible for beneficence [3]. Others advocate for decisions grounded in physician–patient dialogue [4] or the use of predefined treatment goals as reference points [5]. These diverse approaches reflect that determinations of medical futility are not binary or unilateral, but rather deeply complex [6, 7]. While many of these studies have focused on normative frameworks, there remains a need for deeper interpretive analysis of how value conflicts unfold in clinical practice. This paper draws on interpretive phenomenology to explore the ethical conditions under which shared decision-making can be understood as a relational and interpretive practice.

In clinical contexts involving medical futility, the disagreement between physician and patient is often not merely a matter of informational asymmetry but stems from fundamentally different existential structures. Heidegger’s [8] concept of *being-in-the-world* suggests that human existence is always embedded in concrete, historically situated worlds. Each person inhabits a distinct lifeworld shaped by temporality and thrownness. His analysis of temporality reveals that patients’ decisions “in the moment” are inseparable from their lived past and anticipated future. From this perspective, illness is not simply a neutral biomedical condition but an existential rupture that transforms the patient’s very mode of being. As such, patients should not be reduced to diagnostic categories or prognostic trajectories.

The disagreement over whether Ms. M had entered a stage of medical futility was not based on differences in clinical information, but rather on the disparate existential horizons from which physicians and patients interpreted her situation. This reveals that the ethical challenge of determining futility is ontological in nature: physicians and patients cannot be assumed to share the same interpretive world; rather, they are situated within different trajectories of meaning and concern.

In this context, Gadamer’s [9] notion of the *fusion of horizons* (*Horizontverschmelzung*) offers a philosophical grounding for ethical negotiation. For Gadamer, understanding does not occur through the mere transmission of information but through dialogical engagement between divergent perspectives. While complete alignment of values may be impossible, mutual understanding can emerge through a sincere effort to enter the other’s lifeworld. This interpretive fusion is what enables SDM to function not merely as a procedural technique but as an ethical praxis.

From this hermeneutic perspective, judgments of medical futility should not be grounded solely in standardized clinical criteria, but rather understood as ongoing interpretive negotiations. To explore this claim, this study applies Interpretative Phenomenological Analysis (IPA) to examine the narratives and value frameworks of Ms. M and her three attending physicians. Through interviews and textual analysis, the study identifies three interrelated themes:

1. Entering the Horizon—The Patient as a Whole Person Being Seen;
2. Fusion of Horizons—From the Right Decision to the Good Decision;
3. Practicing Shared Decision-Making—A Hermeneutic Framework.

These findings illuminate the ethical tensions inherent in clinical communication and offer a humanistically grounded framework for understanding decision-making in contemporary medicine.

## Methods

To address the ethical tensions arising from the clinical judgments surrounding medically futile treatment, we adopted an interpretive approach grounded in lived experience, rather than relying solely on abstract principle-based dichotomies (such as physician vs. patient, or expertise vs. autonomy). Our rationale is that while the act of treating illness involves objective scientific judgment, the one who is treated is a subjective being—an embodied, feeling, and meaning-making person. As Heidegger emphasized, individuals exist in fundamentally different modes of being, and thus their responses and decisions will necessarily vary.

We therefore employed IPA, a methodology rooted in the phenomenological and hermeneutic traditions, especially influenced by the thought of Heidegger and Gadamer. At the heart of IPA lies an inquiry into how individuals interpret and make sense of significant experiences—such as illness, loss, or decision-making—within the concrete context of their lifeworld [10]. This approach emphasizes context, lived experience, and the interpretive interaction between researcher and participant. Our goal was to explore a space of mutual understanding and connection through semi-structured interviews with both patient and physicians.

IPA entails a two-stage interpretative process:

In the first stage, we focused on how participants interpreted their own experiences, emphasizing a faithful rendering of their narratives. We drew on Heidegger’s notion of *Dasein* [11]—the human mode of being as always already situated in a meaningful world (*being-in-the-world*). Heidegger’s notion of *Geworfenheit* (“thrownness”) refers to *Dasein*’s already-being-in a world of factical conditions not of one’s own choosing—bodily states and disease trajectories, family and social roles, language and culture, religious commitments, institutional norms, and resource constraints. Thrownness is not a one-off surprise but an ontological structure of “having-to-be in” such conditions. Clinically, both clinicians and patients act within this facticity: clinicians inherit ongoing trajectories under guidelines, time pressures, and resource allocation; patients’ viable options are shaped by comorbidities, financial capacity, caregiving duties, and value commitments. SDM must first acknowledge this thrown condition and then work with *projection* (*Entwurf*)—what can still be chosen—within these limits and their ethical implications. Consequently, what counts as a clinician’s “right decision” may not be the patient’s “good decision,” because the latter must remain livable within the patient’s factical conditions.

Heidegger also recasts *temporality*: human existence is not lived in abstract, linear time but in a temporal unity where past experience and future projection shape present understanding. For patients, decisions made in the present are not merely responses to current illness; they are drawn by memories of suffering and unfinished life projects while oriented toward an anticipated future. This temporal structure gives each clinical choice existential weight and requires SDM to take this broader “already–toward” horizon into account.

Following Aho’s [11] interpretation, we understood Dasein not as a fixed identity but as a process of becoming, continually shaped by illness, temporality, and relationality. Accordingly, we used semi-structured interviews to explore how participants made sense of illness within the situated contexts of their lives [10]. As Conrad [12] argues in his concept of “illness as experienced,” patients’ subjective experience of illness carries its own epistemic and ethical value. Attending to the insider perspective allows us to understand how illness reshapes a person’s life and self-understanding.

The second stage moved toward what Gadamer [9] terms the “*fusion of horizons*,” engaging in a dialogical interpretation of participant narratives to uncover embedded values and existential meanings. This stage foregrounds the positionality and interpretive responsibility of the researcher—how to attend to structure, context, and ethical complexity without distorting the data. We paid particular attention to meanings that emerged through language, as well as to implicit existential claims that might remain unspoken yet nonetheless crucial to healing. This resonates with Morgan’s [13] idea in narrative therapy of “alternative stories,” which often reside behind dominant illness narratives. In this framework, the goal of interpretation is not to enforce consensus of values but to locate possibilities for mutual understanding and ethical recognition in the face of difference.

### Supplement: operationalizing the double hermeneutic

In practice, we followed the double hermeneutic central to IPA. Step 1 focused on participants’ sense-making: memos were written immediately after interviews, and transcripts were repeatedly read to attend to words, metaphors, tones, and silences. Step 2 entailed the researcher’s interpretive engagement: using Gadamer’s *fusion of horizons* as systematic perspective-taking, alternating between patient and physician horizons, and testing emerging interpretations through dialogical questions. For example, we reflected on how, within certain institutional or risk contexts, a physician’s decision might be understood as reasonable and responsible, or how, in another clinical horizon, a patient’s choice might be seen as reconstructing meaning. After each shift, transcripts were revisited to check wording, tone, and silences, avoiding over-interpretation; tensions were noted as disconfirming cases and used to revise interpretations. Themes crystallized not through mechanical coding but through hermeneutic engagement, memo-writing, and horizon-shifting.

In addition, the researcher’s positionality significantly shaped the interpretive process. The primary author was trained in philosophy, with a specialization in medical ethics, and has taught medical humanities and medical ethics in a medical school for over a decade, with a long-standing research focus on physician–patient relationships. The researcher has also conducted 14 in-clinic observations and more than 40 interviews with physicians and patients, which sensitized her to the ethical tensions and existential meanings embedded in clinical contexts. At the same time, she recognized that such a philosophical orientation might incline her to privilege the patient’s existential perspective. For instance, when analyzing Ms. M’s recollection of Hospital A’s refusal to perform surgery, she initially perceived the response as cold. Through reflexive journaling, however, she deliberately sought to inhabit the physician’s standpoint, reflecting on how such a decision might be intelligible within professional and institutional contexts. This horizon-shifting did not directly yield conclusions at the methodological stage but served as an important catalyst for the later development of themes. Reflexive writing and continual checking against the transcripts were used to mitigate the risk of over-projecting her own perspective and to revise initial assumptions when tensions arose. This positionality was thus both a resource and a methodological challenge, and the analysis sought to remain transparent and self-disclosing.

### Participants and data collection

1. IPA emphasizes idiographic depth and interpretive specificity, often with small sample sizes [10]. We therefore selected Ms. M as a focal narrative case. Her cervical cancer progressed from stage I to terminal within seven months, during which she experienced two sharply contrasting clinical environments and decision-making styles between Hospital A and Hospital B. Her perspective provides valuable insight into why patients may persist in treatment despite a medical consensus of futility, thereby contributing to our understanding of patient reasoning within shared decision-making.
2. The three attending physicians at Hospital B—specializing in cardiology, cardiac surgery, and gynecologic oncology—offered complementary perspectives. From the standpoint of evidence-based medicine, withholding further treatment may appear objectively justified. Continued intervention, by contrast, is more ethically complex and harder to justify. That these physicians chose to persist in treatment, despite recognizing its likely ineffectiveness, offers a window into the plurality of ethical meanings assigned to futility in clinical practice.

All interviews were approved by the institutional ethics committee (IRB). Informed consent was obtained from all participants, including permission to use their anonymized data for research and publication. Interviews were conducted face-to-face and lasted approximately 25–40 min. Given IPA’s emphasis on the depth of lived experience, interview prompts were directionally designed and implemented as semi-structured conversations. Eighteen questions were asked in total—ten for the patient and eight for the physicians—focusing on clinical decision-making, emotional response, value considerations, and communicative dynamics. An additional table provides full questions (see Additional file 1).

It should be noted that any physician–patient dialogues cited in the Results & Discussion reflect Ms. M’s recollected retelling during the interview, rather than verbatim transcripts of the clinical encounter. This distinction aligns with a hermeneutic approach, in which participants’ interpretive framings are considered meaningful data in their own right.

## Results & discussion

Given the hermeneutic orientation of this study, data analysis was not a linear process but involved a constant movement between transcripts, researcher reflexivity, and theoretical dialogue. Therefore, we present the Results and Discussion together. Each theme is illustrated with excerpts from participants, followed by interpretive analysis and engagement with relevant literature and philosophical frameworks, demonstrating how the themes emerged within the clinical and ethical context.

From the participants’ language, narratives, and interactions, we identified three core themes that reveal the underlying motivations and ethical reasoning behind why both patients and physicians chose to pursue continued treatment, even in situations defined as medically futile.

### Theme 1 : entering the horizon—the patient as a whole person being seen

In the process of participating in shared decision-making, patients often long to be seen as a “whole person”—that is, to have their lived experiences, values, and existential circumstances fully understood and acknowledged [14]. This notion of “being seen” goes beyond visual recognition; as Gadamer [9] emphasizes, understanding is an event—one that only arises when a subject is willing to open their horizon and incorporate the other’s situatedness. When clinicians are able to genuinely listen to and comprehend the existential appeal embedded in the patient’s narrative, the therapeutic relationship can become an enactment of care. As Cassell [15] famously stated, “It is the person who suffers, not the body.” Medical practice, therefore, should respond not to pathophysiological data alone but to the suffering subject.

During the interview, Ms. M generally maintained a composed and determined tone in recounting her illness. Yet she wept twice when recalling the response of a physician at Hospital A regarding “futility of treatment” and, later, the caring actions of a physician at Hospital B before surgery.

We return to Ms. M’s account to examine the significance of these tears. The first episode concerns her recollection of Hospital A, where the physician considered further aggressive treatment futile and suggested a transition to palliative care:

In the interview, Ms. M recalled asking her physician, *“Does this mean I don’t even have the chance for surgery?”*

She recounted that the physician responded: *“Yes*,* there is a chance. But if you undergo surgery*,* it would be in vain. I would not perform it unless something happened and you were brought to the emergency room.”*

While recalling this moment, Ms. M’s tone became tense and she began to choke up. She added: *“When he said that*,* I felt they were so cold. How could a doctor say something like this to a patient? … I felt as though they had given up on me! I… I wasn’t untreatable. I was just harder to treat.”*

Her tears, triggered by the memory, reveal the deep psychological impact of that exchange. Ms. M was not denying her prognosis. Rather, what pained her was the emotional experience of abandonment—the sense of being excluded from care. As Kleinman [16] points out, the biomedical category of disease cannot encompass the social, cultural, and existential suffering of illness. Physicians should not reduce the patient’s illness problem to a disease problem. Toombs likewise insists that the body is not merely a collection of organs but the very ground of the patient’s experience of the world: “I am embodied not in the sense that I have a body — as I have an automobile, a house, or a pet — but in the sense that I exist or live my body.” [17] Patients long to be seen not merely as bearers of symptoms, but as whole beings in specific life situations. Medical practice should thus be understood not simply as a technical intervention, but as an ethical and relational encounter.

The second episode concerns Ms. M’s recollections of her interactions with a cardiologist after being referred to Hospital B.

In the interview, Ms. M recalled: *“His clinic hours were already over*,* but he still stayed with me in the consultation room*,* waiting for my test results… I really felt that I had finally found a good doctor. He truly cared about me…”*.

Ms. M emphasized this point, indicating that such acts of accompaniment carried profound symbolic meaning for her. It revealed how, in the course of illness, the experience of “being cared for” and of “entering the physician’s horizon” could carry far greater value than clinicians might assume. She went on, through tears, to recount her preoperative experience:

Ms. M recounted: *“The night before the surgery*,* he came to see me in the ward. He said he wanted to pray for me. It was the first time anyone had ever offered to pray for me… I really… I cried so hard after he left… He gave me so much confidence*,* because I had been really afraid. I had never undergone heart surgery before; this was my first time.”*

What touched her most was not the religious gesture itself but the physician’s presence and compassion. It was a mode of *being-with* that affirmed her existence as a person. In that moment, Ms. M felt seen in her totality—not only her diagnosis, but her fears and her longing for connection. The physician’s care extended beyond clinical treatment and offered psychological and existential healing. This sense of relationship was grounded not in technical performance, but in emotional resonance and mutual recognition.

Her tears were not merely emotional release; they pointed to something more fundamental—her need to be seen and treated as a person worthy of care and companionship.

Our findings suggest that in shared decision-making, patients are more likely to trust and accept recommendations when they feel the physician genuinely cares about their safety and well-being. When physicians are able to stand alongside patients and provide emotional support, the patient’s strength and confidence may be amplified far beyond expectation [18]. While the physician at Hospital A acted from a risk-management perspective, with the patient’s best interests in mind, the Hospital B physician chose instead to begin with Ms. M’s existential situation—emphasizing understanding and presence.

As Charon [19] has argued, affective care is foundational to the trust that sustains the clinical encounter. Schneiderman and Jecker [20] likewise note that while treatments and therapies may be futile, care never is. They remind us that medicine is not only the execution of technical interventions—it is the enactment of care, relationship, and moral engagement. This insight invites us to ask a deeper question: in making clinical decisions, are we pursuing the right decision, or the good decision?

### Theme 2: fusion of horizons—from the right decision to the good decision

Extending the patient’s desire to be seen as a whole person, we argue that once the physician enters the patient’s horizon of understanding, a further ethical movement must take place: the fusion of horizons between physician and patient, which enables a genuinely collaborative clinical decision. In this process, the ethical priority should be placed on achieving a *good decision* rather than merely a *right decision*.

Although the medical literature provides a classification of futile treatment—such as physiologic futility, quantitative futility, and qualitative futility [21, 22]—these frameworks often expose, rather than resolve, value conflicts in clinical settings. In particular, they rarely clarify the precedence or interplay between physiologic and qualitative futility. As Lemmens [6] and Muller & Kaiser [7] have argued, clinical decisions based on rigid, universal standards often overlook the significance of patients’ life stories and subjective values.

The present case illustrates precisely this difficulty. The same patient who was deemed a candidate for futile care at Hospital A later received a successful heart surgery and ongoing care at Hospital B. This divergence cannot be explained by medical criteria alone; rather, it reveals differing ethical perspectives and interpretive horizons. Hospital A made a *right decision* based on empirical risk assessment and prognosis guided by EBM principles. In contrast, Hospital B responded to the patient’s expressed values and hopes, attempting to shape a *good decision* from within the patient’s lifeworld.

This leads us to a deeper question about the nature of medical decision-making: Are we striving for the *right decision*, grounded in statistical efficacy and clinical safety, or the *good decision*, grounded in ethical responsiveness to the patient’s subjectivity, relationality, and existential reality? While in some cases the *good* and the *right* may converge, in others they may diverge and even come into tension. As Pellegrino [23] emphasizes:

“Each participant is a moral agent and as such is bound to uphold, and be accountable for, his, or her, own conception of what is right and good. Making morally defensible decisions in the face of substantive differences in conceptions of patient good has become, therefore, one of the urgent procedural problems in medical ethics.”

Recent scholarship in medical ethics has further developed this idea. Daniel Sulmasy [24] insists that good clinical decisions must be grounded not only in scientific knowledge but also in the patient’s existential and relational context. Pellegrino and Thomasma [25] similarly propose that medical goodness involves an integration of technical correctness, ethical appropriateness, and personal values. This view reframes medicine not simply as a professional task but as a fundamentally ethical response to another human being.

In this study, the statements of the three physicians at Hospital B reflect attempts to engage in such an ethical response, shaped by the fusion of horizons with the patient:

The cardiologist remarked: “Even if this surgery is just to help her face the end of life properly, I think it’s worth it.” — reflecting a commitment to the integrity of life’s final stage.

The cardiovascular surgeon said: “I mostly considered the medical side… If she is going to take on the risk of surgery, what benefit will that risk bring her?” — pointing to the need for proportionate benefit in terms of quality of life.

The gynecologic oncologist observed: “If a cancer patient can live another three or six months, that’s worth it to us.” — emphasizing the existential significance of time extension.

Though these perspectives seem different on the surface, they all represent ethical efforts to interpret treatment choices from within the patient’s horizon. Each physician moves beyond the logic of the right decision and toward the ethical imagination of the good decision. As Gadamer argues, understanding is not a matter of consensus but of the expansion that arises through the fusion of differing horizons. Physicians do not abandon their clinical expertise, but they step into the patient’s lived world to encounter her hopes, fears, and search for meaning.

Fusion of horizons does not ask physicians to surrender their expertise but invites them to enlarge their ethical vision. Clinical decisions should not be governed solely by data and risk models but should also attend to the moral and affective life of the patient. In this sense, decision-making becomes an ethical dialogue, not merely a technical procedure.

Gadamer [9] further critiques modern scientific thought for severing our experience from its historicity, misleading us into believing that standardized data can be universally applied. Yet every patient is a unique *Dasein*, a being with a story, context, and temporality.

This insight recalls the ancient Greek view of medicine as an art (*techne*). Plato [26] warned:

“You should not attempt to cure the eyes without the head, or the head without the body, nor the body without the soul.”

This holistic view is ever more urgent today, when medical technology can prolong physiological life but often at the cost of existential suffering. Callahan [27] warns of the danger of “a cruel kindness” when life is sustained at the expense of meaning.

Thus, the designation of “futile treatment” should not be dictated unilaterally by clinicians through EBM, but approached as an open, negotiated, and interpretive process with patients. While physiologic futility may offer technical reference points, qualitative futility better approximates our ethical vision of whole-person care.

Ms. M’s case illustrates that her choice to undergo surgery was not grounded in denial of her condition but in her desire to fulfill unresolved wishes, mend relationships, and die on her own terms. For some, an extra three months may mean little. For her, it offered a space for meaning, dignity, and closure.

In such cases, the clinical question is not simply “Is this treatment effective?” but rather: “Is this meaningful to her?”

### Theme 3 : practicing shared decision-making—a hermeneutic framework

Although Evidence-Based Medicine (EBM) provides a critical scientific foundation for modern clinical decision-making, its logic of “averaging” often fails to accommodate the unique situations of individual patients [28]. The issue of “medical futility” examined in this study vividly illustrates this tension, as such determinations frequently involve a complex entanglement of physiological, psychological, ethical, and existential dimensions [29]. Given that patients—not physicians—are the ultimate bearers of medical decisions [7], judgments about what constitutes “futile” treatment cannot rely solely on scientific objectivity; they must also attend to the patient’s existential situation as a whole being. Only when physicians and patients achieve mutual understanding of each other’s horizons can Shared Decision-Making (SDM) fulfill its ethical promise.

The difficulty of determining medical futility often stems from a lack of communication and mutual understanding between physicians and patients [30]. Therefore, integrating EBM with what we term a value-based medicine(VBM) approach is essential to optimizing SDM. The concept of “Value-Based Health Care” (VBHC), introduced by Porter and Teisberg [31] and further elaborated by Porter [32], emphasizes maximizing patient outcomes relative to costs. In contrast, our use of “Value-Based Medicine” refers to a hermeneutic framework that foregrounds the patient’s existential, ethical, and narrative dimensions, thereby complementing the predominantly physiological focus of EBM.

**Fig. 1:**
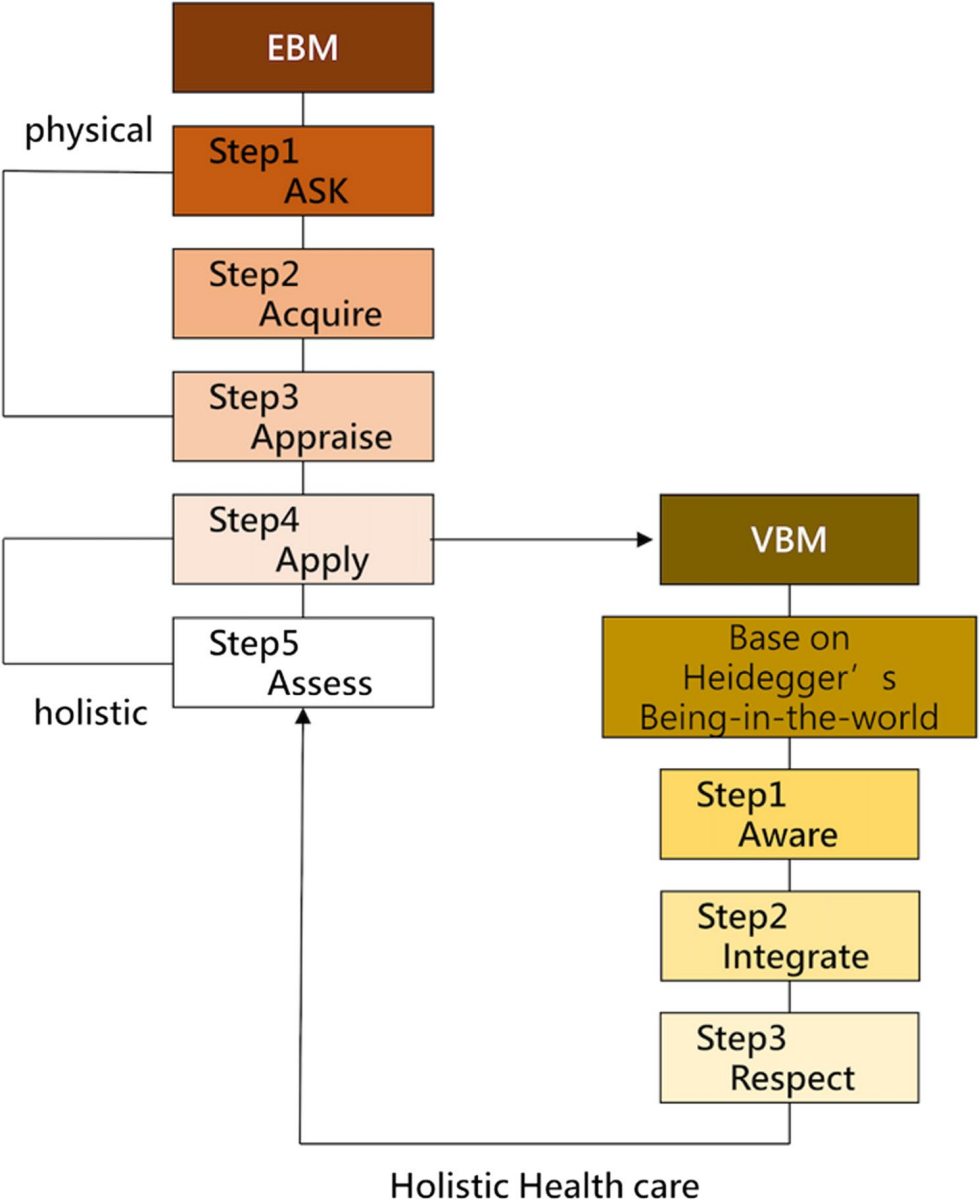
An integrated model of evidence-based medicine (EBM) and value-based medicine (VBM) in shared decision-making

According to the five-step model of EBM proposed by Guyatt et al. [33], the first three steps (Ask—Acquire—Appraise) primarily focus on the patient’s physiological dimensions. The final two steps (Apply—Assess), however, should be expanded to incorporate the psychological and existential dimensions of the patient. Only through such a holistic assessment can true holistic health care be achieved (see Fig. 1).

The left side of Fig. 1 illustrates the five-step framework of EBM, while the right side presents our proposed VBM model, developed within a hermeneutic framework grounded in Heidegger’s concept of Being-in-the-world and Gadamer’s notion of the fusion of horizons. Unlike VBHC in the policy literature, our VBM emphasizes the holistic and existential dimensions of patient care, thereby complementing the predominantly physiological emphasis of EBM.

#### Step 1: aware

The concept of *horizon* refers to the individual’s pre-structured way of understanding the world, shaped by cultural background, lived experiences, beliefs, and historical context. As Gadamer [9] argues, horizons are not closed systems; rather, they are capable of being expanded and merged through dialogue. In the initial stage of SDM, physicians must first become aware of the differences between their own and the patient’s lifeworlds and recognize the potential gaps between these horizons. For example, from a clinical risk perspective, a physician might view a procedure as futile due to its high risk, while the patient may see it as their last opportunity to repair meaningful relationships or complete unfinished life tasks.

#### Step 2: integrate

The fusion of horizons does not require the physician to abandon their own value commitments; instead, it calls for building a bridge of understanding across differences through deep listening, ethical dialogue, and empathetic engagement. The physician should strive to understand *why* the patient is making a particular decision at a particular moment, rather than merely evaluating the choice through biomedical criteria. In our study, physicians at Hospital B respected Ms. M’s value-laden decision to undergo a high-risk surgery, even though it did not align with EBM-defined optimal options. They recognized that “for her, this was a journey of coexisting with death and reconstructing life’s meaning.” This form of understanding emerged not through persuasion or authority, but through a hermeneutic response grounded in the fusion of horizons.

#### Step 3: respect

When complete overlap of horizons cannot be achieved, physicians should nonetheless prioritize what constitutes a *good decision* from the patient’s perspective. This does not entail abandoning professional responsibility; rather, it involves acknowledging that the goal of medicine is not merely the extension of biological life, but the realization of meaningful existence. Physicians remain responsible for providing accurate information and assisting in decision-making, but not as instrumental executors of the patient’s will. In this way, SDM can integrate the *rightness* of EBM with the *goodness* of the patient’s decision, avoiding the instrumentalization of the physician’s role [34] and alleviating the moral distress that arises in cases of futile care [5].

These three steps are not mechanical procedures, but ethical postures and hermeneutic practices. While the five steps of EBM (Ask, Acquire, Appraise, Apply, Assess) focus predominantly on physiological and evidentiary dimensions, our SDM model emphasizes humanistic understanding. Only when physicians are willing to enter into the patient’s horizon—and when patients genuinely feel understood—can medical knowledge be transformed into practical wisdom. As Marcel reminds us, the true aim of medicine should concern not merely the concept of a thing, but the thing itself, that is, the *Being* of the person.

Through VBM, we affirm the structural wholeness of the patient’s existence and draw upon the philosophical perspectives of Heidegger and Gadamer to enrich the ethical practice of SDM. Furthermore, we advocate for strengthened training in ethical dialogue within medical education—such as courses in narrative communication—so that future physicians may develop the capacity to enter the patient’s horizon, discern value conflicts, and engage in meaningful conversations. In doing so, the clinical space can be transformed from a site of technical intervention into one of mutual healing and the co-construction of ethical meaning.

Furthermore, when value differences exist among members of the healthcare team, interprofessional communication and consensus-building are equally essential. The plurality of medical perspectives should not be seen as a source of conflict but as an opportunity to promote integrative and holistic patient care. It is through engaging such differences that medical teams can critically reflect on what truly constitutes *the good in medicine*.

## Conclusions

This study underscores that Evidence-Based Medicine (EBM) and our hermeneutic interpretation of Value-Based Medicine (VBM) should not be treated as dichotomous or mutually exclusive frameworks, but rather as complementary approaches that together construct a patient-centered model of Shared Decision-Making (SDM). Importantly, our conception of VBM differs from “Value-Based Health Care” (VBHC), as it places greater emphasis on the existential dimensions of patient care.

At the same time, this study has important limitations. As it focuses on a single patient case alongside interviews with several physicians, the findings are not intended to be generalized across all clinical contexts. Instead, they highlight how IPA and hermeneutic analysis can illuminate the ethical and existential stakes of medical futility, pointing to dimensions of meaning that might otherwise remain implicit.

We argue that the best medical decision must reconcile the *rightness* of EBM with the *goodness* of the patient’s decision. The practice of SDM requires physicians to demonstrate not only ethical sensitivity but also interpretive competence, moving beyond a technical model of “offering options” toward an ethical interaction of “co-constructing meaning.” For this reason, we call for medical education and clinical training to cultivate physicians’ capacities for ethical dialogue, narrative attentiveness, and existential understanding. Only when physicians can enter the patient’s lived horizon and engage in genuine co-interpretation can SDM be realized not merely as a procedural tool, but as an ethical practice and an expression of humanistic wisdom. 

## Supplementary Information

Supplementary Material 1.

## Data Availability

The datasets generated and/or analyzed during the current study are not publicly available due to ethical restrictions but are available from the corresponding author on reasonable request.

## Abbreviations

SDM: Shared decision-making
EBM: Evidence-based medicine
VBM: Value-based medicine
IPA: Interpretative phenomenological analysis
