# Supplementary material for: Medical futility and the ethics of continuing treatment: a hermeneutic inquiry into patient and physician perspectives
Source: Philos Ethics Humanit Med. 2025 Oct 21;20:33. doi: 10.1186/s13010-025-00200-3 (PMC12539090; doi:10.1186/s13010-025-00200-3)
Supplement: Supplementary file 1 — Supplementary Material 1. [file 13010_2025_200_MOESM1_ESM.docx]

# Appendix 1. Semi-Structured Interview Guide

## A. Patient Interview Questions (n = 10)

| Question No. | Thematic Focus | Interview Prompt |
| --- | --- | --- |
| 1 | Illness Trajectory | Can you describe your experience from the time you were diagnosed to the present? |
| 2 | Emotional Experience | What were your greatest fears or anxieties during this process? |
| 3 | Decision-Making Process | How did you make medical decisions? Did you consult anyone? |
| 4 | Influence of Others | Whose opinions most influenced your choices? |
| 5 | Emotional Reflexivity | How did you feel when making your final decision? |
| 6 | Understanding of Info | Were the physicians’ explanations clear to you? How did you deal with uncertainty? |
| 7 | Communication Experience | How would you describe your communication with the physicians? |
| 8 | Values and Concerns | What factors influenced your medical choices (e.g., family, finances)? |
| 9 | Expectations of Care | What kind of help or support did you most hope to receive from your doctor? |
| 10 | Social Support | How did you hope people around you would support you during illness? |

## B. Physician Interview Questions (n = 8)

| Question No. | Thematic Focus | Interview Prompt |
| --- | --- | --- |
| 1 | Clinical Encounter | Can you describe your first meeting with this patient and your treatment plan? |
| 2 | Initial Impressions | What impression did the patient make on you at first? |
| 3 | Perceived Needs | What needs or pressures did you perceive the patient to have? |
| 4 | Treatment Considerations | What factors did you consider when making treatment suggestions? |
| 5 | Emotional Responses | How did you feel emotionally while guiding the patient through decision-making? |
| 6 | Communication Barriers | Did the patient fully understand your explanations? How was the communication? |
| 7 | Professional Challenges | What was the most difficult part of caring for this patient? |
| 8 | Retrospective Reflection | In retrospect, is there anything you would have done differently? |
